# The legacy of *Drosophila* imaginal discs

**DOI:** 10.1007/s00412-016-0595-4

**Published:** 2016-05-07

**Authors:** Jorge V. Beira, Renato Paro

**Affiliations:** 1 Department of Biosystems Science and Engineering, ETH Zürich, Mattenstrasse 26, 4058 Basel, Switzerland; 2 Faculty of Science, University of Basel, Klingelbergstrasse 50, 4056 Basel, Switzerland

**Keywords:** Drosophila, Imaginal discs, Patterning, Regeneration, Tumor model

## Abstract

The study of *Drosophila* imaginal discs has contributed to a number of discoveries in developmental and cellular biology. In addition to the elucidation of the role of tissue compartments and organ-specific master regulator genes during development, imaginal discs have also become well established as models for studying cellular interactions and complex genetic pathways. Here, we review key discoveries resulting from investigations of these epithelial precursor organs, ranging from cell fate determination and transdetermination to tissue patterning. Furthermore, the design of increasingly sophisticated genetic tools over the last decades has added value to the use of imaginal discs as model systems. As a result of tissue-specific genetic screens, several components of developmentally regulated signaling pathways were identified and epistasis revealed the levels at which they function. Discs have been widely used to assess cellular interactions in their natural tissue context, contributing to a better understanding of growth regulation, tissue regeneration, and cancer. With the continuous implementation of novel tools, imaginal discs retain significant potential as model systems to address emerging questions in biology and medicine.

## Introduction

The initial discovery and molecular characterization of numerous gene products, known to play key roles in human physiology and medicine, were first described in fruit flies. Indeed, detailed genetic analyses in *Drosophila* revealed when and where many genes are required for developmental and cellular processes, and thus furthered our understanding of conserved molecular mechanisms. The amenability of *Drosophila* to carry out genetic screens, given the short generation time and considerable number of progeny, remains one of the main advantages of this model organism. While many genes have been identified through embryonic mutant screens, the characterization of critical gene function at different developmental stages has substantially benefitted from studies in imaginal discs. These structures are epithelial tissues that develop during the earlier stages of the life cycle in holometabolous insects and ultimately give rise to major adult body parts such as eyes, wings, legs, or genitalia.

Imaginal discs have provided a useful platform for studying fundamental aspects of biology, mainly due to their accessibility and the development of ingenious methods to manipulate the genetic content of cell populations within discs. Critically, the shared similarities of disc cells with the epithelial cells that protect most human organs highlights the relevance of discoveries made with imaginal discs for biomedical research (Jennings 2011; Wangler et al. 2015). The use of imaginal discs as experimental systems overcomes the limitations of lethal embryonic mutations, because patches of mutant tissue can be generated and analyzed at later developmental stages. Indeed, significant technical advances have contributed to these achievements, mainly due to targeted or tissue-specific expression of a construct of interest (with the GAL4/UAS system) and its possible combination with the generation of homozygous mutant clones within a wild-type tissue (with Flp/FRT) (Brand and Perrimon 1993; Chou and Perrimon 1992, 1996; Golic and Lindquist 1989).

This review is part of a series celebrating the work of Walter Gehring. He was first exposed to the biology of imaginal discs as a graduate student with Ernst Hadorn in Zurich, where he studied the capabilities of antennal imaginal discs to change fate (transdetermine). A desire to elucidate the mysteries of antenna-to-leg reprogramming observed in these early disc transplantation experiments and his concomitant identification of a *Drosophila* homeotic gene phenocopying this particular transformation provided the driving force behind his outstanding research career. This eventually led to the molecular characterization of the *Antennapedia* gene and the groundbreaking discovery of the homeobox, providing the basis for a new concept in developmental biology and highlighting extraordinary evolutionary conservation at the molecular level. Throughout his career, Walter Gehring treasured the advantages of imaginal discs for studying developmental processes and his legendary skills in genetically manipulating or transplanting discs inspired many scholars to continue the use of this exceptional experimental paradigm.

Here, we review a number of key discoveries made possible through experiments in imaginal discs as well as describe current topics and active research areas that benefit from discs as experimental systems. We bring together a wide range of fundamental concepts and discoveries, which are usually kept disconnected in topic-centered reviews, to ultimately showcase the importance of imaginal discs. We aim to provide an integrated perspective that connects key discoveries, such as cell determination, transdetermination, the homeobox, and the genetic control of development. We build upon these topics to further connect conserved signaling cascades functioning during normal development and regeneration to how their derailment permits tumor initiation. We also provide a succinct overview of genetic methods and clever tools available for disc manipulation, which have recently permitted sophisticated experiments for elucidating basic concepts of tissue regeneration and cancer. Discs serve as a canvas to experimentally address how cells and tissues respond to gain or loss of specific gene functions. The potential of using imaginal discs to aid future discoveries remains unchallenged, thus promising further contributions in cell and developmental biology.

## The development of imaginal discs and their embryonic origin

In order to fully appreciate the contributions of imaginal discs and their lasting impact on modern biology, it is important to first provide some background about their development from embryonic tissues. During development, many organisms first develop miniature versions of their adult body structures, which eventually increase in size. However, *Drosophila* undergo substantial morphological changes during the life cycle, forming precursor structures during earlier molting stages that will not simply grow but be substantially transformed during metamorphosis. Adult flies have well-developed appendages (eyes, wings, legs, halteres, and genitals), while larvae require less complex structures for simple behaviors such as feeding or foraging. The structures that will give rise to external appendages in the adult remain protected within the larva. These precursor structures are referred to as “imaginal,” as they will give rise to the adult body structures known as the “imago.” Imaginal structures are not limited to the epidermal sac-like cell clusters known as discs but also include histoblast nests, which will form the abdominal epidermis, and other small groups of cells of the gut or salivary glands (Cohen 1993; Lawrence 1992).

**Fig. 1:**
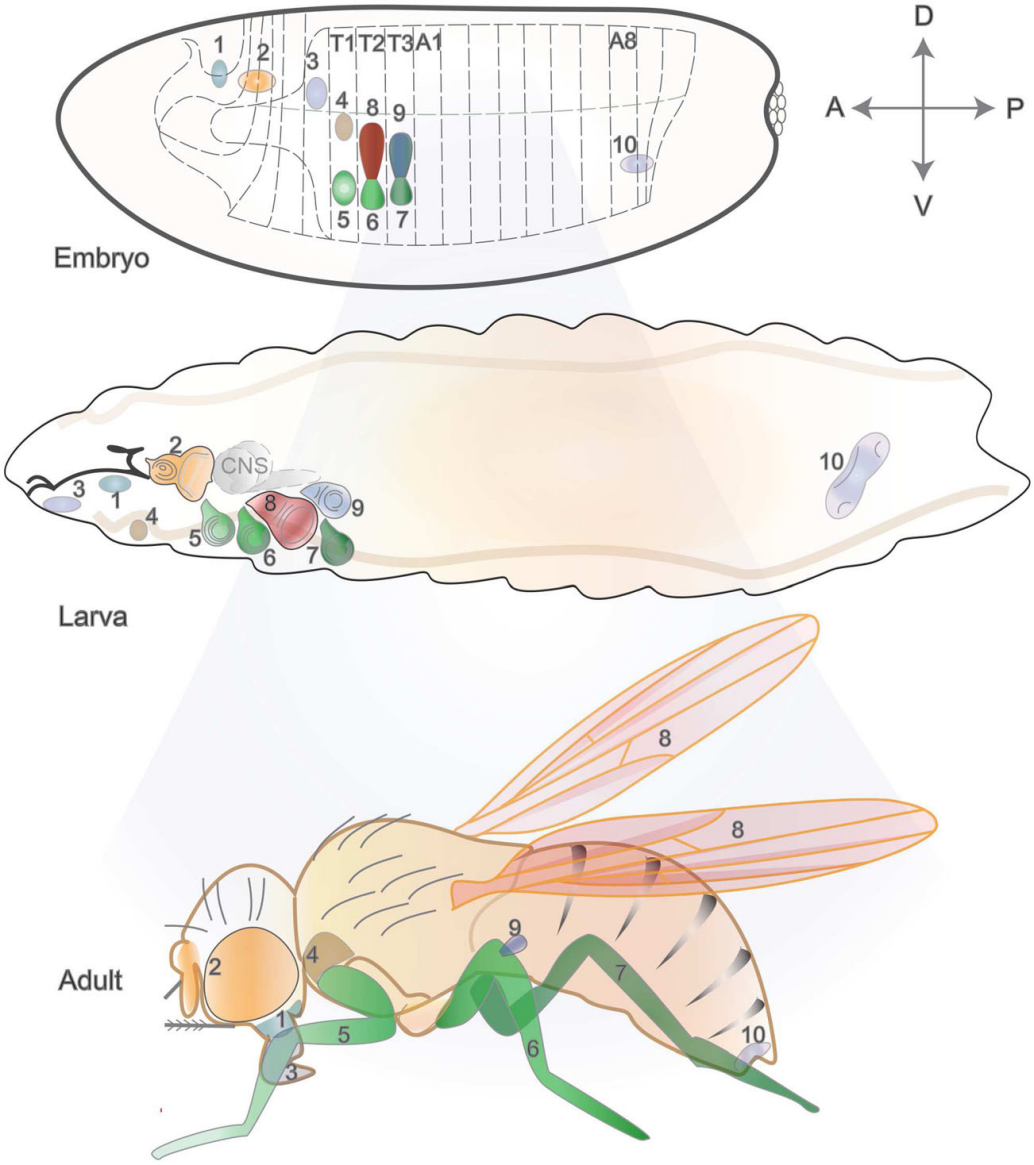
Imaginal discs, their embryonic primordia, and adult cuticular products. The location of imaginal tissue primordia is represented at the cellular blastoderm stage (*top*), with corresponding numbering in larval (*middle*) and adult (*bottom*) stages. Axes orientation is indicated by the *perpendicular arrows* (*A* anterior, *P* posterior, *D* dorsal, *V* ventral). *T1* to *T3* represent thoracic segments, and *A1* to *A8* correspond to abdominal segments. The epidermis of adult structures like the head, thorax, and appendages come from 9 pairs of bilateral discs (here, only one of each pair is shown in the larva), and genitals derive from a middle disc (19 discs in total): *1*, clypeolabral; *2*, eye-antennal; *3*, labial; *4*, humeral (or prothoracic); *5*, first leg; *6*, second leg; *7*, third leg; *8*, wing; *9*, haltere; *10*, genital. Note that some portions of the head and thorax, including the notum, also originate from imaginal discs. For instance, the wing discs contribute both to the wings and the notum in the adult fly, which is not represented here for simplicity. Parts of the figure were inspired by (Held 2005), where additional details are described

In total, there are 19 discs in the larva, with nine bilateral pairs that will form epidermal structures, and a genital medial disc (Held 2005) (Fig. 1). Labial and clypeolabral discs will form the mouthparts. Eye-antennal discs will give rise to the compound eye and the antenna, and are in close contact with the mouth hooks and the optic lobes of the central nervous system. The three pairs of leg disc primordia arise in the embryo from the ventral ectoderm, one pair per thoracic segment (first-leg discs in T1 and second- and third-leg discs in T2 and T3, respectively) (Cohen 1993). Each thoracic segment also produces a pair of dorsal imaginal discs, namely humeral (or dorsal prothoracic), wing, and haltere discs. Finally, the genitalia arise from a medial disc spanning abdominal segments A8 to A10 and are sexually dimorphic, thus having distinct morphology and growth in males and females (Chen and Baker 1997; Estrada et al. 2003; Held 2005; Sánchez and Guerrero 2001).

Each imaginal disc arises from a cluster of few cells in the embryo, and the morphology matures during larval stages. The embryonic epidermis is formed by epithelial cells, which have a characteristic apico-basolateral architecture that is key for their function. The apical domains of disc cells face the lumen, and cell-cell adhesion enables epithelia to serve as a protective barrier. Disc experiments have formed our understanding of the protective barrier function of the epithelia, since they surround most organs across species. Imaginal discs become sac-like structures upon invagination from the embryonic ectoderm. Discs have an outer layer, the peripodial membrane, with squamous cells that provide little contribution to the cuticular structures in the adult (Haynie and Bryant 1986; Held 2005). The disc proper is formed by a single columnar epithelial layer, which confers advantages for imaging and analysis of structural changes in tissue and cell shape upon manipulations. Most discs also contain some adepithelial cells (mesodermal myoblasts), as well as tracheal cells and a few neurons that all reside between the epithelium and the basal lamina. The genital disc is the only case where cells from the mesoderm are recruited into the epithelium (Held 2005). In a newly hatched first instar larva (about 24 h after egg laying, AEL), the larger discs (wing, leg, and eye-antennal) contain about 20–70 cells (Madhavan and Schneiderman 1977). By mid-to-late first instar, disc cells resume mitosis and continue dividing exponentially during second and third instar stages (Cohen 1993; Nöthiger 1972). Notably, a considerable number of cells appear during the third instar, with cell number doubling about every 10 h. Prior to pupariation, each disc contains from 10,000 to 50,000 cells (Johnston et al. 1999; Morata and Ripoll 1975).

Mature discs undergo a major morphogenetic event during metamorphosis, as they evert through their stalk in a process that is triggered by the ecdysone hormonal cascade (Fristrom and Fristrom 1993; Poodry 1980). Recent imaging and culturing methods have opened the path to document this process using live imaging of fluorescently labeled portions of the wing disc. The dynamics of such morphogenetic movements have been described and include a 90° folding of the disc followed by a rapprochement with the pupal epidermis and finally the disintegration of the peripodial membrane by apoptosis (Aldaz et al. 2010). Whole-tissue imaging further refined this process by following groups of fluorescently marked cells through the epidermis of the developing larva, avoiding the need for dissection (Kanca et al. 2014). This brief overview of imaginal disc development sets the basis for the main purpose of revisiting key discoveries with discs that had a lasting impact in modern biology and across species. For a more comprehensive overview of imaginal disc development, see for example (Cohen 1993; Held 2005).

## From fate mapping to transdetermination

Because the origins of imaginal discs can be traced back to specific positions in the embryo, this experimental system has historically been useful for fate mapping. More specifically, experimental embryologists observed that localized damage to the embryo could have an effect on specific structures of the adult (Gehring 1972; Postlethwait and Schneiderman 1973). Furthermore, disc primordia from embryo fragments could be cultured in vivo, transplanted to larval hosts, and, upon metamorphosis, grow to their approximate normal size and show normal spatial patterns (Schubiger et al. 1969). Studies by Ernst Hadorn and colleagues established imaginal disc transplantation techniques, which led to the creation of fate maps and provided information about the organization of the mature disc (Hadorn 1965; Schubiger and Schubiger 1978; Schubiger et al. 2012). The cell potential (or determination) was assessed by the variety of structures that would arise from the transplanted tissue, indirectly revealing cell fate restriction or plasticity.

The subject of cell determination received considerable attention in the pre-molecular era and benefitted greatly from the burgeoning field of molecular biology. Genetic studies showed that discs originate as groups of founder cells, known as polyclones (Crick and Lawrence 1975). Garcia-Bellido and Merriam used gynandromorphs (flies that are sexual mosaics, composed of male and female tissues due to the loss of one X chromosome during early cleavages) to ask if discs originated from a single cell, having only one sex. This was not the case, and together with transplantation studies using eye-antennal, leg, wing, haltere, and genital discs, it became clear that imaginal discs are of multicellular origin (Garcia-Bellido and Merriam 1969; Wieschaus and Gehring 1976). Transplantation of single cells from the embryo tested the possibility of reprogramming of cellular fate according to the location where they would be placed, yet cells could retain their parasegmental identity following transplantation. Transplantation of beta-galactosidase-marked cells from a blastoderm stage embryo tested the contribution of clones originating from injected cells. These were found to contribute to both larval and imaginal structures, demonstrating that no lineage restriction exists at this stage and, thus, that imaginal identity is established later (Vincent and O’Farrell 1992). These experiments also resolved issues about tissue compartments, as described in the next section.

**Fig. 2:**
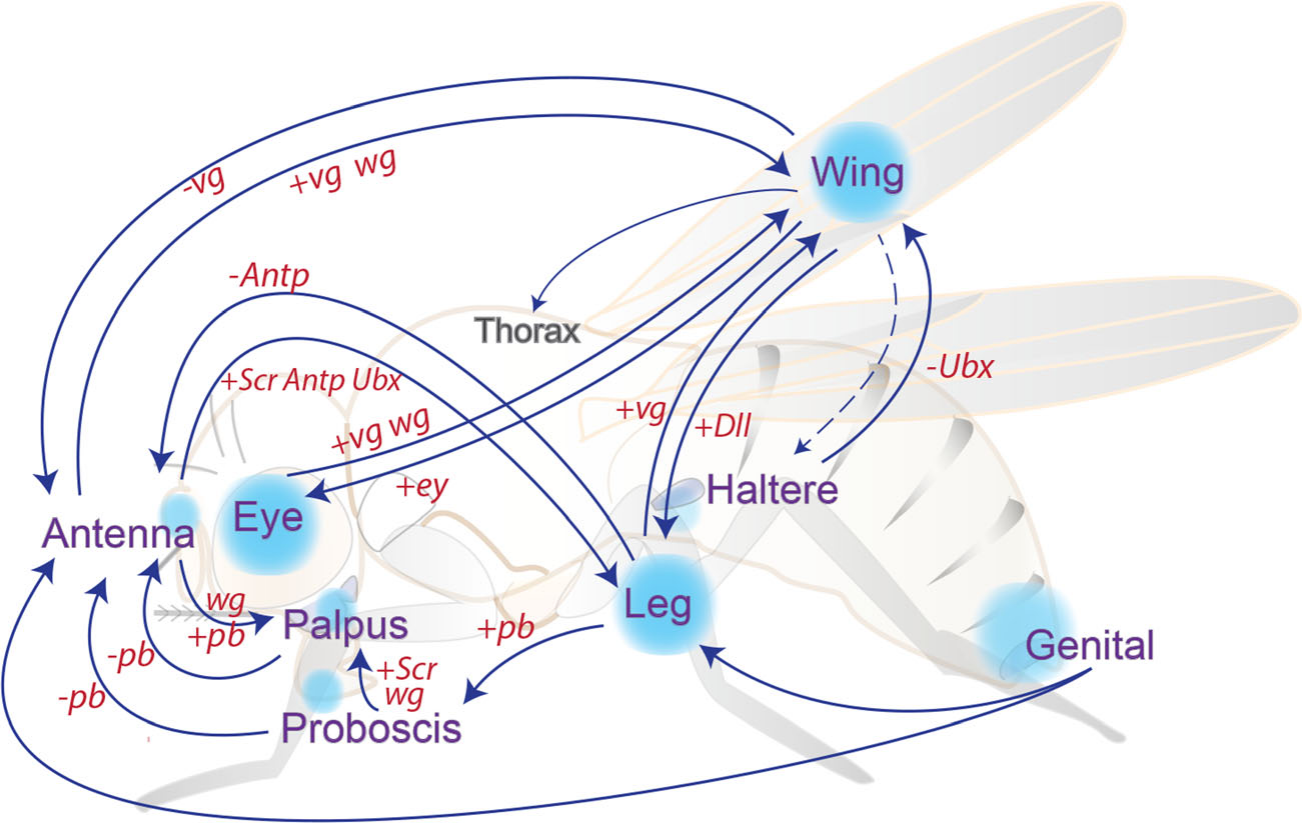
An overview of transdetermination events, resulting both from disc fragmentation and through genetic manipulations. Classical transdetermination events occur with some probability upon disc fragmentation and culture through transplantation, some with higher frequencies like leg-to-wing (see main text). Ectopic expression of *wg* (*wingless*) also results in transdetermination in situ, similarly to what seems to occur at “weak points,” where endogenous high levels of Wg and Dpp have been linked with switches in cell fates. Genetic manipulations also recapitulate transdetermination of some fly tissues into distinct ones (*arrows connecting the blue circles*), specifically by gain (+) or loss (−) of homeotic or selector gene expression (*Ubx*, *Ultrabithorax*; *Antp*, *Antennapedia*; *pb*, *proboscipedia*; *Scr*, *Sex combs reduced*; *ey*, *eyeless*; *Dll*, *distal-less*; *vg*, *vestigial*). The figure was inspired by Wei et al. (2000) and McClure and Schubiger (2007)

While performing serial transplantation experiments, Hadorn witnessed an unexpected observation: despite transplanting genital discs, he noticed the later appearance of ectopic antennal tissue as a result of a process which was then termed *transdetermination* (Gehring 2002; Hadorn 1965; Nöthiger 2002). This propensity was later shown to occur with other discs displaying eye-to-wing or leg-to-wing transformations. Moreover, it was realized that cutting discs through sensitive regions yielded transdetermination events at higher frequencies (Fig. 2) (Schubiger 1971; Worley et al. 2012). Some of the molecular players involved in transdetermination are similarly crucial for the regenerative process in wounded tissues, and thus, a parallel has been suggested between the molecular mechanisms regulating transdetermination and regeneration (Bergantiños et al. 2010b) (as discussed in more detail below). Experiments based on disc fragmentation and transplantation contributed to addressing issues of cell determination and transdetermination. These initial studies provided a basis for branching questions, such as cellular plasticity, regeneration, or maintenance of cell identity.

## Compartments and patterning signals in imaginal tissues

The concept of tissue patterning lies at the core of understanding organ development, since it provides the initial cues necessary for genetic cascades to produce shape and function at later stages. The basic rules underlying patterning and the organization of the body plan were revealed as a result of combining molecular biology techniques with genetics, a step forward that considerably expanded developmental genetics. Sequential activities of gene networks establish individual segments along the anterior-posterior axis of the fly embryo. In turn, these patterning signals are integrated with instructions from *Hox* genes whose downstream targets include morphogens like *wingless* (*wg*), *hedgehog* (*hh*), or *decapentaplegic* (*dpp*) (Jaeger 2011; Lawrence and Struhl 1996; Morata 2001; Scott and Carroll 1987). Individual segments will thus develop distinct features, resulting from differential transcriptional programs in response to a specific combination of signals. While imaginal disc patterning differs in some aspects from anterior-posterior patterning in embryonic segments, several players are common to both stages and throughout appendage development.

At the molecular level, presumptive disc cells in the embryo already express genes like *distal-less* (*Dll*), *vestigial* (*vg*), *escargot* (*esg*), and *snail* (*sna*) that will later be required not only for normal disc development but also at later stages (Cohen et al. 1993, 1991; Cohen 1990; Ray et al. 1991; Whiteley et al. 1992; Williams et al. 1991). For instance, ventral (leg) discs initially form in the ventral ectoderm and can be identified by expression of the homeotic gene *Dll*. Whereas the presumptive dorsal disc cells (wing and haltere) express *vg*, and these discs invaginate from the epidermis during dorsal closure (Cohen 1993; Williams et al. 1991). The embryonic cells that will give rise to thoracic imaginal discs are stereotypically located where the expression of *wg*, *dpp*, and *engrailed* intersects (Cohen et al. 1993). The expression of *dpp* in the embryo is perpendicular to that of the segment polarity genes *wg* and *engrailed* (*en*), a feature that is also observed, for example, in the wing disc. *Engrailed* is expressed in the posterior region of both embryonic segments and imaginal discs, where it not only functions as a selector gene but also defines tissue compartmentalization, a concept further examined below. It became clear that anterior-posterior (A-P) boundaries in imaginal discs reflect earlier lineage restriction and are initially established as parasegment boundaries in the embryo (Dahmann and Basler 1999; Martinez-Arias and Lawrence 1985).

The discovery of tissue compartments was revealed by clonal analysis, which also proved very fruitful for fate mapping and lineage tracing. Compartments are the result of a lineage restriction between the anterior and posterior portions of the wing disc (Garcia-Bellido et al. 1973; Morata and Lawrence 1975). Such restriction occurs early in the *Drosophila* embryo, where all segments contain two separate lineages, anterior and posterior, which are segregated and form a stable boundary throughout development. For instance, when clones are genetically induced, they can arise in either compartment, but they do not cross from one to the other. The concept of tissue compartments had broader implications for understanding development in other organisms, since they were later found to similarly exist in the developing chicken hindbrain and the mammalian brain (Dahmann and Basler 1999; Fraser et al. 1990; Ingham and Arias 1992; Levitt et al. 1997).

The molecular mechanisms underlying allocation of cell fates among anterior and posterior identities were found to require a key gene, *engrailed* (Morata and Lawrence 1975). Clonal experiments uncovered a role for *en* in the selection of posterior identity. Clones lacking *en* in the posterior compartment develop into anterior structures and no longer respect the boundary, while mutant clones arising in the anterior compartment develop normally. Furthermore, an ectopic boundary formed in the P compartment at the interface between wild-type posterior cells and *engrailed* mutant clones, which behave as anterior cells (Lawrence and Struhl 1996; Morata and Lawrence 1975). These results from clonal analyses, together with the observation that wings of adult flies lacking *en* exhibit transformation to anterior structures, clarified the role of *en* as a selector gene. In addition to the A-P compartment boundary, a second tissue division appears during larval stages, at the dorsal-ventral (D-V) border. In the case of this orthogonal subdivision, the selector gene *apterous* was identified to establish the differences in dorsal-ventral identities and also to play a role in wing growth and patterning (Diaz-Benjumea 1993).

**Fig. 3:**
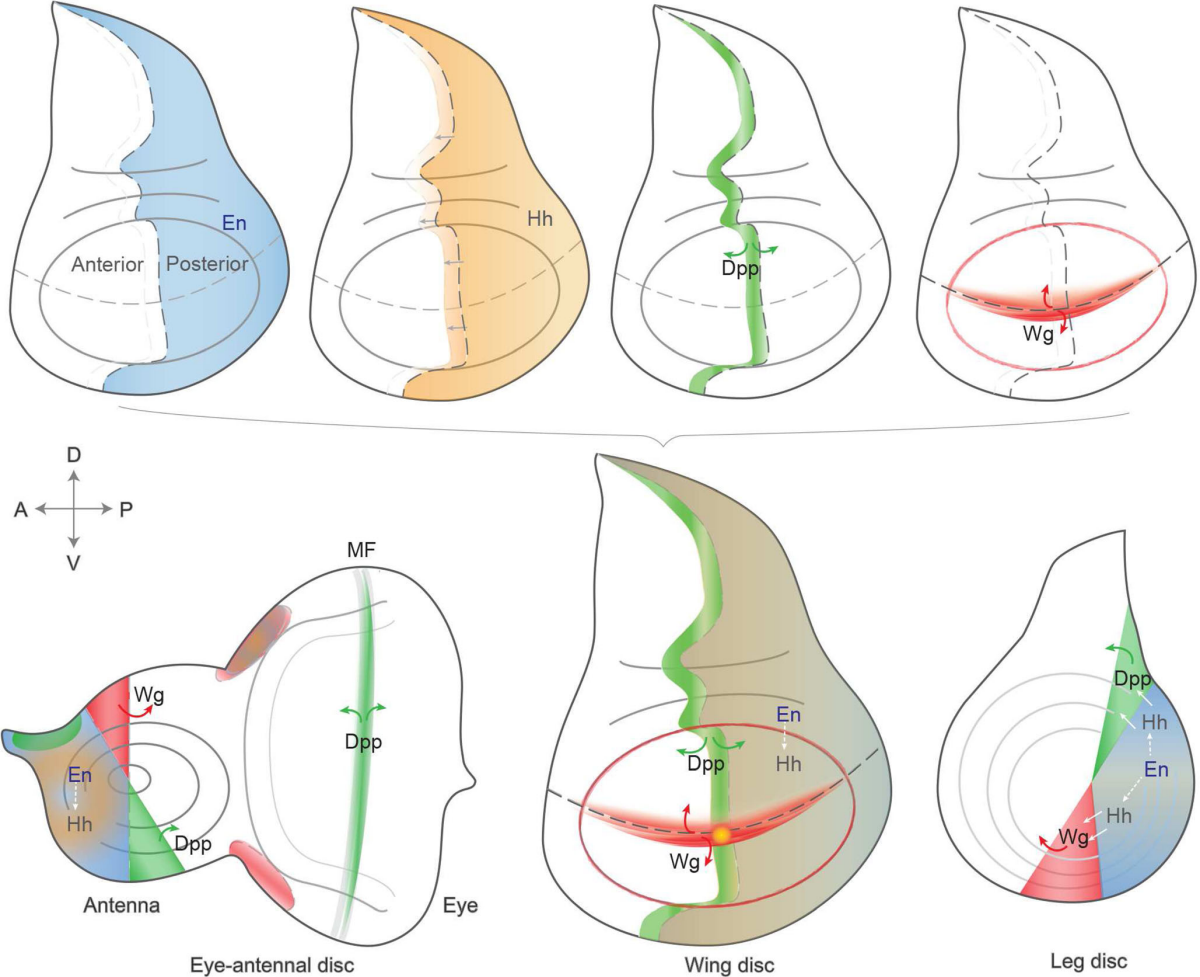
Expression domains of key signaling and patterning pathways in wing, eye-antennal, and leg discs. Imaginal discs become subdivided and patterned during development, under the concerted action of signaling pathways and morphogens. Four key signals are represented individually in the wing disc (*top*). Engrailed (*en*) is expressed in all cells in the posterior (*P*) compartment, conferring posterior identity and thus establishing the anterior-posterior (*A-P*) boundary. En directs expression of the secreted short-range signaling molecule, Hedgehog (*Hh*), which can cross the A-P boundary and induce expression of Decapentaplegic (*Dpp*). Dpp is expressed along the A-P boundary, and its secretion permits long-range signaling to direct patterning of a wider disc region. Wingless (*Wg*) is produced at the dorsal-ventral (*D-V*) boundary, a signal that is also key for wing development (see main text). The signaling domains are represented all together in the bottom central wing disc, and their roles are also similarly conserved in other tissues, like the eye-antennal (*bottom left*) and leg (*bottom right*) discs. A similar color coding is used in all discs, which are oriented with anterior to the left and dorsal up. Expression patterns at other developmental stages are described in Held (2005)

The wing imaginal disc proved ideal to uncover the molecular relationships between patterning regulators, as within this, tissue morphogens play a role in both the A-P and D-V axes, which intersect perpendicularly in the pouch region. The same cascades also function in other discs, as illustrated in Fig. 3. Engrailed inhibits Hedgehog signaling in the posterior compartment, but Hh secretion permits short-range signaling and thus regulates key genes like *wg* (encoding a Wnt family member) or *dpp* (a homolog of the TGFβ family of growth factors) in neighboring cells such as to activate the expression of *dpp* in a stripe next to the A-P boundary. The identification of several components of these signaling cascades revealed conserved signal transduction pathways, having an impact on physiological and cellular functions across species. For an overview on the patterning roles of these three pathways and their morphogen properties, see Tabata and Takei (2004).

Importantly, regulation of compartment boundaries is linked to the establishment of novel proximal-distal signaling axes in insect appendages, leading to the activation of proximal selector genes that confer appendage identity, such as *vestigial* and *scalloped* in the wing. The pioneering research connecting compartment boundaries to the proximal-distal patterning axis in fly imaginal discs has illuminated important aspects of vertebrate limb development as many basic rules operating in this system are shared in distantly related organisms (Brook et al. 1996; Dahmann et al. 2011). For a comprehensive description of the molecular determinants involved in appendage development, see for example Mann and Morata (2000) and Morata (2001).

The functional relevance of tissue compartments became more apparent with the finding that the domain of action of some homeotic genes was also restricted. Homeotic transformations refer to alterations of specific body segments or structures and are the phenotypic manifestation of mutations in homeotic genes. For example, a spontaneous homeotic mutation led to transformation of the anterior portion of the third thoracic segment (aT3) into the anterior portion of the second (aT2) and was named *bithorax* (*bx*, later found to be an allele of *Ubx*). Another mutation with a complementary effect was also isolated, *postbithorax* (*pbx*), where the posterior part of T3 (pT3) is transformed into pT2. The combination *of bx* and *pbx* mutations by meiotic recombination enabled Ed Lewis to produce the famous four-winged fly, where a second pair of wings develops instead of halteres, showcasing a functional consequence of mutations in the bithorax complex (BX-C) (Lewis 1978). For a comprehensive discussion of the bithorax complex, see another review in this series (Maeda and Karch 2015). Another homeotic transformation that was identified, among several, was *Antennapedia* where legs develop instead of antennal structures, thus naming a second *Hox* gene complex, ANT-C (Schneuwly and Gehring 1985).

The realization that many genes implicated in development and cell fate decisions contained a homeobox established a shared principle for regulation relying on transcriptional changes. More explicitly, the combinatorial activities of homeobox genes result in distinct cell fates in specific tissues and organs (Struhl 1982). The strict regulation of *Hox* gene expression in specific segments or compartments proved to be at the core of a faithful developmental program, as evidenced by homeotic phenotypes resulting from their dysregulation. The maintenance of ON or OFF states of homeobox genes in specific expression patterns was found to depend on Trithorax (TrxG) and Polycomb group (PcG) genes, respectively (Ringrose and Paro 2007). Although initially studied for their embryonic phenotype, their function is also crucial for cellular memory in imaginal discs, as early studies showed for some genes that are regulated by PcG/TrxG, like *en*, *hh*, or *wg* (Ingham 1983; Maurange and Paro 2002; Paro and Hogness 1991; Randsholt et al. 2000). Molecular analyses made clear that PcG and TrxG proteins form multimeric complexes involved in epigenetic regulation, especially as they contain enzymatic activities responsible for catalyzing histone modifications, H3K27me3 by *E(z)* or H3K4me3 by *Trx* (Beisel and Paro 2011; Byrd and Shearn 2003; Czermin et al. 2002; Müller et al. 2002). PcG/TrxG target several hundred genes (most of which are developmental regulators) and play a global role in chromatin regulation and genome architecture that is conserved in many species (Boyer et al. 2006; Schwartz et al. 2006; Sexton et al. 2012; Tolhuis et al. 2006). Thus, the *Hox* gene clusters were used as a starting point to identify the underlying regulatory mechanisms, resulting in far broader implications for global genome function and chromatin biology, a field that is currently sprouting aided by new methodologies.

## Studies on organ growth and shape, cellular interactions, and signaling cascades

**Fig. 4:**
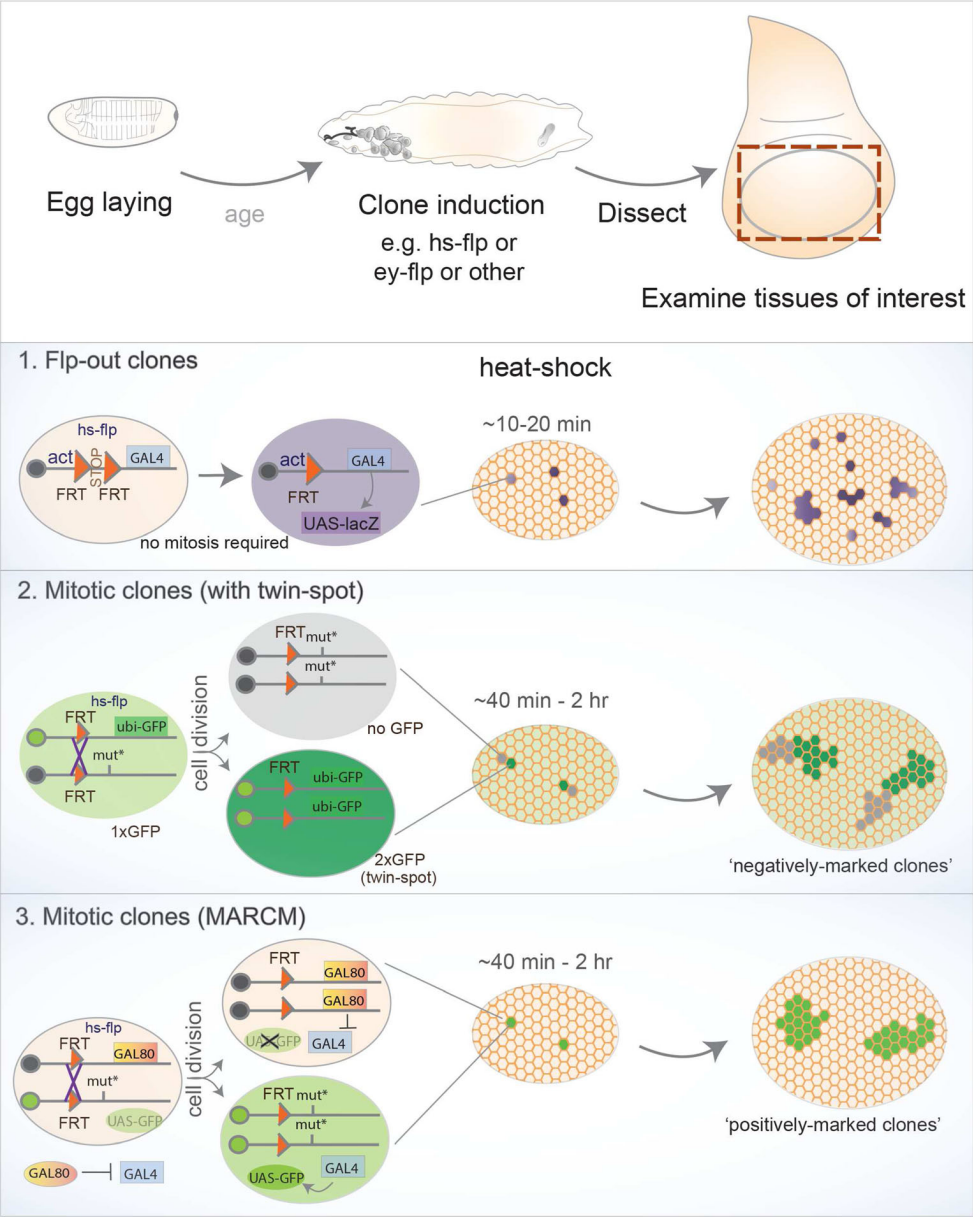
Overview of clone-generating techniques. Three broadly used techniques relying on the Flp/FRT system, where the source of Flippase can be chosen depending on experimental design, e.g., commonly using the *heat shock* promoter with temporal control or tissue-restricted promoters (*ey*, *Ubx*). *Top*: after egg laying for a defined period and aging larvae to the desired stage, mosaics are induced upon Flp expression and, by dissecting the tissues of interest, the effect of genetic manipulations is analyzed by comparing clones with neighboring wild-type tissue. (*1*) Flp-out clones do not rely on mitosis, since Flp mediates recombination of two FRT (Flippase-Recognition Targets) elements with the same orientation located in close proximity in one chromosome. Usually, a constitutive promoter (actin, tubulin, etc.) is not active in the absence of Flp due to an element flanked by FRT sites (e.g. stop, or *yellow* marker). Excision of the flanked element upon Flp-dependent recombination enables the promoter to activate a downstream gene only within the clone (for example, a marker such as lacZ, Gal4 (that could activate a UAS-lacZ), or other alternatives). (*2*) Mitotic clones require cell division, where somatic recombination of chromosomes in heterozygous cells provides the opportunity for Flp-mediated recombination. In a given parental cell (heterozygous for a mutation of interest), recombination between matching FRT elements located in homologous chromosomal arms produces two daughter cells: one carrying two copies of the wild-type chromosome and another homozygous for the desired mutation. The following cell divisions will therefore generate two cell populations, hence a clone and its wild-type counterpart. A constitutive marker is often used (e.g., a ubiquitous GFP construct inserted in the wild-type chromosome), and thus the homozygous mutant clone can be identified by the absence of the marker (negatively marked), while the wild-type “twin” resulting from the same cell division (hence called twin spot) can also be visualized as it harbors two copies of the marker (2xGFP). (*3*) An alternative method was developed to enable clones to be marked “positively,” i.e., labeling the tissue harboring the desired genetic manipulation with GFP. This option is provided by MARCM (Mosaic Analysis with a Repressive Cell Marker), where the constitutive expression of a repressive component (Gal80) on the homologous chromosome prevents Gal4 from activating downstream genes or markers (usually UAS-GFP). Upon Flp expression, recombination produces a homozygous mutant cell that lacks Gal80 while the other contains a pair of FRT chromosomes carrying Gal80 (and no mutation). In the mutant clone, the absence of Gal80 permits Gal4 to activate the UAS-GFP marker (and also permits further flexibility as other UAS transgenes can be expressed specifically within clones). In this case, the wild-type tissue remains unlabeled and only mutant clones are visually detected

Alongside the identification of spontaneous mutations in some homeotic genes, screens for mutations affecting specific imaginal discs uncovered several classes of phenotypes, many affecting organ growth or shape (disc undergrowth, overgrowth, or even hyperplastic growth) (Cohen 1993; Shearn and Garen 1974). However, in general, these approaches only identified mutations causing local effects or those not leading to premature lethality because observing the phenotype required survival beyond embryonic stages. To overcome this limitation of whole organism mutations, successful genetic screens combined mutagenesis (with chemical mutagens, like ethyl methanesulfonate (EMS), or P-element-mediated mutagenesis) with genetic mosaic-inducing techniques, such as Flp/FRT-mediated mitotic recombination. Such strategies permitted the characterization of gene function in restricted tissue patches (Chou and Perrimon 1996; Spradling and Rubin 1982; St Johnston 2002) (Fig. 4). Furthermore, clonal analysis enabled discrimination between cell-autonomous and non-cell-autonomous effects to further clarify the importance of cellular interactions between mutant tissue and the surrounding “wild-type” tissue.

Imaginal discs have also been fundamental for characterizing the role of conserved signaling pathways in developing tissues. The amenability for epistasis experiments revealed the function of several pathway members in vivo and at what cascade levels they work. In addition to the main patterning signals mentioned before (Wg, Dpp, and Hh), fly research has also been at the root of discoveries spanning many members of conserved signaling cascades such as Notch, EGFR (epidermal growth factor receptor), MAPK (mitogen-activated protein kinase), JAK-STAT (Janus kinase and signaling transducer and activator of transcription), JNK (Jun N-terminal Kinase), among others.

Defective eye specification revealed roles for transcription factors like *eyeless*, *twin of eyeless* (*toy*), *eyes absent* (*eya*), *eyegone*, *sine oculis*, and also Notch and EGFR signaling acting upstream of this transcriptional network (Kumar 2001). Interestingly, many members of this gene network responsible for the eye-antennal transcriptional program contain a homeobox, which is a recurring feature of transcription factors acting during development. Enhancer and suppressor screens revealed a number of components acting within the same pathways. For example, epistasis experiments clarified the role of *spitz* as a ligand that can bind to the Egf receptor (*torpedo*), leading to the activation of downstream kinases encoded by *ras* and *raf* and culminating in transcriptional regulation by Pointed (Kumar 2001; St Johnston 2002). Notch signaling can be triggered by ligands, such as Serrate and Delta, binding to the receptor (Notch) which, after cleavage of the intracellular domain by gamma-secretase, can partner with *Su(Hw)* (*suppressor of hairy wing*) to modulate transcriptional activation. For a more complete summary of Notch signaling, see Guruharsha et al. (2012). Importantly, bristle specification and imaginal disc experiments uncovered three Notch activities that established paradigms in neural development across species: cell fate assignment, boundary formation, and lateral inhibition (Bray 1998; Gómez-Skarmeta et al. 2003).

The global signaling rules employed by cells in a tissue are key to understanding how cells communicate with their immediate neighbors or even how distant intercellular interactions play a role in normal physiology. The in vivo function of a considerable number of newly identified signaling components was achieved through clonal or genetic experiments in *Drosophila* (which goes beyond the scope of this review) (Hynes et al. 2013; Jenny and Basler 2015; Kumar 2001). One of the central questions regarding the integration of different signaling instructions through cellular interactions concerns how growth control is achieved. Growth regulation has attracted considerable attention in the last decades and thus deserves some mention, as several genetic screens have identified regulatory genes.

Wg and Dpp are instrumental in wing disc patterning and growth, and have been classified as morphogens since both proteins can spread further away from the cells where the respective genes are expressed (Vincent and Briscoe 2001). It has been proposed that morphogen gradients are responsible for instructing growth and patterning of the wing disc and may have similar functions in vertebrates (Ashe and Briscoe 2006; Tabata 2001). For example, the Dpp gradient could lead to activation of distinct targets depending on the distance from the source (the A-P boundary), as suggested by the nested expression patterns of its target genes. Three well-characterized targets are *spalt major* (*salm*), *optomotor blind* (*omb*), and *brinker* (*brk*). While *salm* expression is centered around the source, the domain of *omb* expression is broader than *salm*, and *brk* is expressed at higher levels at the periphery, forming an opposing gradient to *dpp* (Affolter and Basler 2007; Campbell and Tomlinson 1999; Nellen et al. 1996). Moreover, ectopic *dpp*-expressing clones could produce duplications of wing veins with correctly patterned territories (Capdevila and Guerrero 1994; Zecca et al. 1995). Similarly, the evidence for the existence of a Wg gradient was also supported by nested expression domains of targets described along the D-V boundary, e.g., *senseless* (*sens*, a high-level target), *distal-less* (*Dll*, with a more extended range), and *vg* (a low-level target expressed in most of the prospective wing) (Neumann and Cohen 1997; Zecca et al. 1996). However, recent evidence challenges the requirement of a Wg gradient since flies carrying solely an engineered version of *wg* that is unable to spread develop wings with nearly the right size and without apparent morphological defects (Alexandre et al. 2014). It has been suggested that cells expressing *wg* at earlier stages maintain expression of target genes due to a cellular memory mechanism, even after the inducing signal is absent. Regarding Dpp, the role of this morphogen in tissue patterning and growth has attracted considerable attention, and a concentration-dependent response has been put forward to account for differences in size by affecting cell proliferation (Vuilleumier et al. 2010; Wartlick et al. 2011). Two recent studies, taking advantage of endogenous genome editing methods, suggest that the Dpp gradient is required for patterning but not essential for cell proliferation in lateral portions of the wing disc, thus contributing to growth mostly in the pouch region (Akiyama and Gibson 2015; Harmansa et al. 2015). All these reports showcase a topic of intense research that also has an impact beyond *Drosophila*, and thus, it will be interesting for future studies to determine how these observations and models will fit together.

Indeed, the wing disc became an established model for the study of morphogens and how they spread in tissues. For example, analogous patterning functions for Sonic Hedgehog (SHH, a homolog of Hh) were uncovered during mammalian neural tube development (Kicheva et al. 2012). Moreover, mathematical modeling where physics concepts were applied to biological data from the wing Dpp gradient could generate hypotheses about gradient properties, and an “expander” protein (encoded by *pentagone*) has been suggested to enable scaling control (Ben-Zvi et al. 2011; Hamaratoglu et al. 2011). These examples demonstrate a variety of outcomes spanning several fields, as a result of a fundamental developmental question, yet with broad implications that are relevant across subjects and model systems.

Although Wg and Dpp (and the components of both pathways) assembled significant attention concerning growth regulation, alternative routes were taken to identify additional genes involved in these mechanisms. Genetic screens were carried out, for example, targeting the eye disc, producing large portions of homozygous mutant eye-antennal discs in an otherwise wild-type animal using site-specific recombination only in the target tissue. The eye disc proved a simple platform for such screens, as it does not affect fertility or viability, and phenotypes are easily assessed by comparing the mutant white patches to the wild-type “twin spots” originating from the same mitotic division (Fig. 4). For example, a version of the Flippase recombinase enzyme (engineered from yeast) only active in the eye-antennal disc (eyFlp) was used to induce recombination of FRT (Flippase-recognition target) sites, producing homozygous mutant tissue upon cell division. Screens were thus carried out using this approach with a wide range of mutations and led to the characterization of genes required for normal tissue architecture and growth. Examples of hits include the tumor suppressor kinases *warts*, *salvador*, and *hippo*, the latter giving its name to a novel pathway that can restrict cell proliferation and promote apoptosis (Tapon et al. 2002; Wu et al. 2003; Xu et al. 1995). Other initial screens for abnormal eye development revealed regulators of cell proliferation, like *archipelago* (through *cyclin E*) and homologs of the *tuberous sclerosis complex* (*Tsc1/2*). Likewise, cytoskeletal or nuclear components influencing cell affinity, adhesion, and eye development were also identified (Janody et al. 2004, 2003; Moberg et al. 2001; Tapon et al. 2001).

Sequencing of the fly genome and the discovery of RNA interference (RNAi) led to the generation of genome-wide transgenic RNAi libraries under UAS control, offering an unprecedented potential to precisely carry out selective reverse genetics using tissue-specific Gal4 drivers (Adams et al. 2000; Dietzl et al. 2007). These tools prompted the design of many genetic screens, either in the eye (with GMR-gal4) or wing compartments (en-gal4 or hh-gal4) where the effect of target gene knockdown could be directly compared to an internal control (e.g., the anterior compartment) where gene function remained intact. Such RNAi-based screens expanded our knowledge about further members of previously mentioned pathways (like Wg, Dpp, Notch, Hippo, etc.) and also fine-tuned our understanding of additional processes. For instance, screens uncovered regulators of apoptosis that could rescue small-eye phenotypes induced by downstream pro-apoptotic inducers (e.g., GMR>hid) and compensatory proliferation (Fan and Bergmann 2008; Herz et al. 2006; Mummery-Widmer et al. 2009; Saj et al. 2010; Thompson and Cohen 2006).

The previous examples of hits resulting from screens and clonal analyses in their complex tissue environments highlight the power of *Drosophila* discs as platforms to identify and characterize conserved factors and associated pathways, often for the first time. The foundation of detailed knowledge gathered on the basic biology of imaginal discs places us in an exciting position to further exploit these model systems to address medically relevant topics where developmental states are challenged, like regeneration and tumor initiation.

## From disc transplantation to tissue regeneration

The process of tissue and organ regeneration has attracted curiosity for many decades. Classical examples of remarkable regenerative capacity come from amphibians, which are able to replace large and complex structures like the tail or limbs (Tanaka and Reddien 2011). The potential to build up on the basic knowledge underlying regenerative mechanisms for possible exploitation into medical applications has captivated many. Despite this profound interest, research using these organisms has been limited because few exploratory tools are available. Conversely, *Drosophila* stands at an interesting point for regenerative studies, since it is less complex than amphibians and humans and a wide palette of experimental tools is available.

**Fig. 5:**
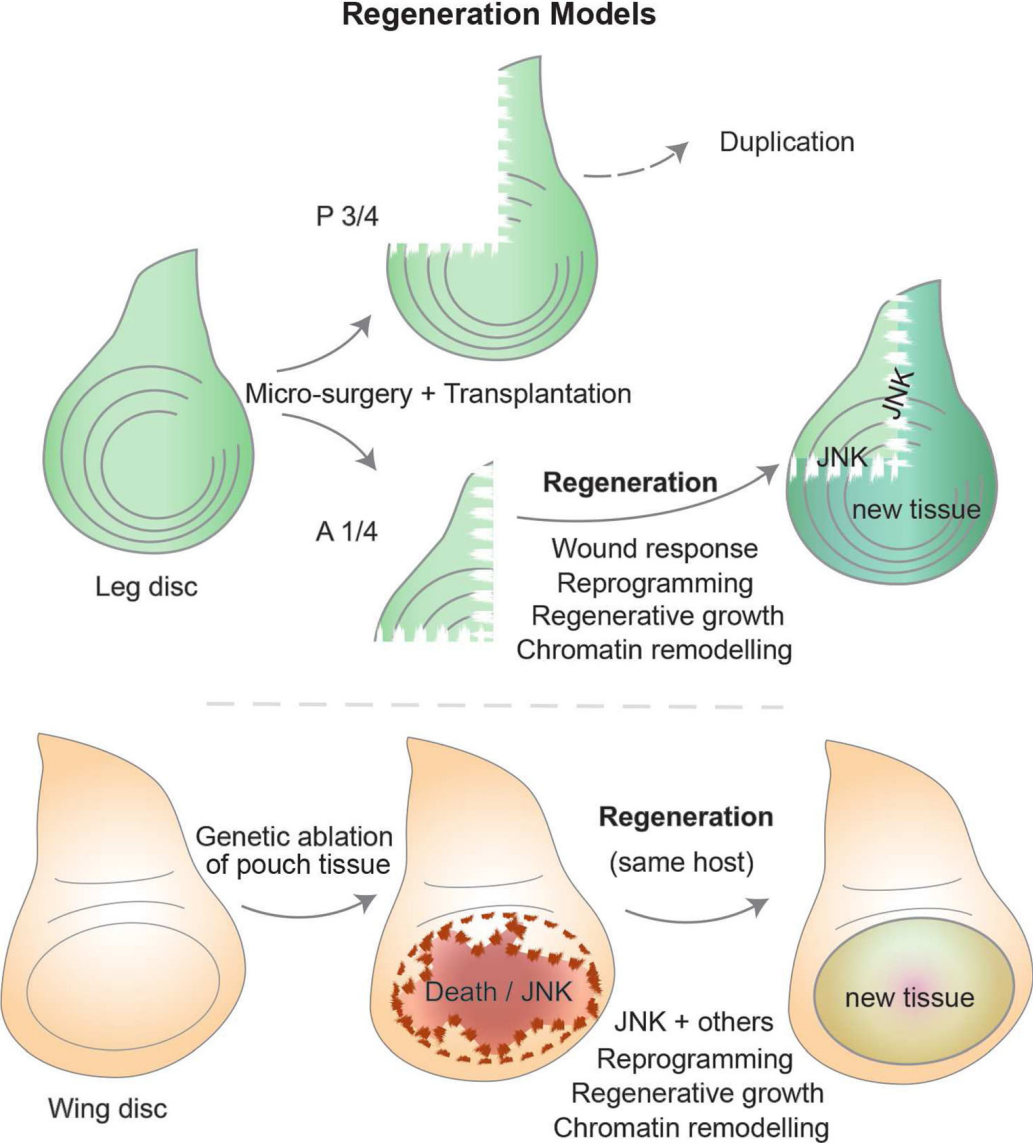
Regeneration in *Drosophila* imaginal discs. Imaginal discs are able to regenerate upon micro-surgery and culture through transplantation to host flies. The leg disc has routinely been used in fragmentation experiments (*top*), often with a standard cut that separates the “anterior one quarter” from the “posterior three quarters.” The larger fragment tends to result in tissue duplication, while the smaller fragment regenerates the remaining tissue with the correct pattern and function. The cut elicits a wounding-like response, followed by a phase of regenerative growth, for which chromatin remodeling is important during cellular reprogramming that can produce extra cells while maintaining the correct pattern and cellular identities. Recently developed tools (*bottom*) allow genetic-induced tissue ablation and regeneration within the same organism (overcoming the limitations of ablation and transplantation). Localized tissue ablation, for example, of the pouch region of the wing disc, can be spatially and temporally controlled. Different systems have been described (see main text), but generally, the design involves the following: a region-specific Gal4 is normally inhibited by ubiquitous Gal80 expression, which can be overcome by means of a temperature shift (with a temperature-sensitive form of Gal80 that is inactive at 29 °C or higher), which duration can be titrated to achieve specific conditions. During the period when Gal80 is inactive, Gal4 can activate a downstream UAS target that triggers apoptosis (e.g., using *rpr*, *hid*, egr, etc.). After an acute or controlled “damage-induction” phase, larvae are returned to a temperature that yields a functional Gal80 and thus inactivates the damage trigger, allowing tissues to regenerate during a “recovery” phase. Many of the hallmarks are conserved between the classical ablation experiments and the genetic-ablation systems, and the recent use of the latter promise considerable advantages that improve reproducibility and larger-scale experiments

The regenerative capacity of fly imaginal discs was noticed several decades ago alongside the transplantation experiments mentioned previously (Bryant 1971, 1975; Hadorn 1965). Upon disc fragmentation, for example, along the D-V axis of the leg disc, the anterior portion can regenerate while the posterior half undergoes duplication where a mirror image of the tissue arises, instead of forming the missing part. Interestingly, the mechanism for this difference still remains a mystery (Bryant 1971; Schubiger 1971). The cells around the cut edges form a blastema containing cells that are able to divide more frequently than in other regions. These fast-proliferating cells are mainly responsible for disc regeneration, although cell death also occurs (Fig. 5) (Abbott et al. 1981; Kiehle and Schubiger 1985). The determination state of the disc seems to be maintained in blastema cells, yet when fragmentation occurs along defined “weak points,” for example, where *dpp* and *wg* overlap, transdetermination events occur with some frequency (Johnston and Schubiger 1996; Maves and Schubiger 1995, 1999). Regeneration studies using disc fragmentation have, therefore, been tied with transdetermination (Worley et al. 2012).

The molecular events required for disc regeneration following fragmentation revealed a role for Wg, as its ectopic expression frequently promoted leg-to-wing transdetermination (Johnston and Schubiger 1996). The molecular basis for transdetermination relies at least in part on interactions between Wg and Dpp signaling, as the activation of *vg* downstream of Wg signaling resulted in the formation of more wing tissue from the dorsal region (where *dpp* is higher) (Maves and Schubiger 1995, 1998). The function of these pathways in blastema cells may facilitate cellular plasticity underlying cell fate re-assignment during regeneration, and parallels with stem cell-like potency have been put forward (McClure and Schubiger 2007; Wei et al. 2000).

The micro-surgery techniques employed to study regeneration, for example, by dissecting the anterior quarter of the leg disc, trigger a wounding response at the early stages of blastema formation. Thus, the early regenerative response utilizes similar molecular routes as wound healing, involving the activation of stress signaling like JNK (Jun N-terminal kinase) (Bosch et al. 2005; Mattila et al. 2005). JNK is crucial at several stages of the regenerative response by directing cytoskeletal rearrangements to bridge the gap between wounded tissues (similar to its function during embryonic dorsal closure (Martin and Parkhurst 2004)), by promoting cell death around the cut edges, and finally by modulating chromatin regulators. The latter function was uncovered as a result of inducing leg-to-wing transdetermination through ectopic *wg* expression using the flp-out clonal technique (Struhl and Basler 1993). This leads to *vg* expression in leg discs, which in turn results in transdetermination to wing tissue, of which frequency is increased upon JNK-mediated overcoming of PcG silencing (Lee et al. 2005). Since Polycomb group (PcG) proteins silence many developmental and signaling-related genes, the involvement of PcG/TrxG links regenerative mechanisms with the regulation of powerful genes that confer tissue identity. Thus, transdetermination events can similarly require a selective bypass of PcG silencing of targets that select a distinct developmental program (as ectopic expression of tissue-specific selector genes can achieve similar results).

The use of modern transcriptomic approaches has revealed additional genes involved in regeneration. For example, *Mmp1* and *puckered* (two targets of the JNK pathway) as well as those activated in response to stress and cell death-related genes become upregulated in regenerating discs (Blanco et al. 2010; Klebes et al. 2005; McClure and Schubiger 2008; McClure et al. 2008). Furthermore, additional chromatin factors were retrieved from transcriptome analyses, namely *lama*, PcG/TrxG, and chromatin remodelers such as the Brahma complex (Blanco et al. 2010; Klebes et al. 2005). The JAK-STAT pathway becomes activated through JNK-dependent upregulation of *unpaired* ligands and has, therefore, also been implicated in regeneration (Katsuyama et al. 2015). Interestingly, the signaling peptide dILP8 was upregulated in cut leg discs undergoing regeneration. Possibly providing the developmental delay required for healing and regeneration, dILP8 is known to induce a delay in when discs are damaged (Colombani et al. 2012; Garelli et al. 2012; Katsuyama et al. 2015).

The use of genetic tools that take advantage of tissue-specific drivers to trigger apoptosis in significant portions of imaginal discs has been employed in recent years to induce cell ablation and subsequent regeneration (Fig. 5). This has been achieved by either expressing effector pro-apoptotic genes, like *hid* or *reaper*, or upstream components that lead to JNK activation in a similar manner to what happens in the blastema (Bergantiños et al. 2010a; Herrera et al. 2013; Smith-Bolton et al. 2009). After a temperature-controlled time window for ablation (using gal80), the regenerative capacity of wing discs and the involvement of key pathways like Wg and Myc was then confirmed during a recovery period (Smith-Bolton et al. 2009).

Such genetic model systems are revitalizing studies on regeneration, since they offer significant advantages over micro-surgery methods, not only improving reproducibility and increasing disc numbers for downstream analysis but also facilitating additional genetic manipulations or even screening. For example, an elegant study has recently revealed that compartment boundaries are transiently broken down, as the cells that contribute to disc regeneration were found in compartments outside of their origin (Herrera and Morata 2014). Fitting with previous observations, PcG and TrxG members also play a role in the process, by modulating the potential to transgress the A-P boundary. As cellular reprogramming has been regarded as an intrinsic aspect of the regenerative process, these results are among the first to test this hypothesis directly. Cell fate re-specification has also been reported, with regenerated veins having contributions from tissue that previously corresponded to inter-veins (Repiso et al. 2013). The reprogramming challenge poses some issues regarding developmental robustness, thus hinting that the regenerative process is likely very tightly regulated ensuring accurate re-patterning and growth. The mechanisms ensuring such control are only starting to be explored, as demonstrated by the recent identification of a factor protecting regenerating tissues from cell fate changes. This is encoded by *taranis*, which regulates posterior cell fate during regeneration but seems dispensable in normal development (Schuster and Smith-Bolton 2015).

Several open questions remain in the regeneration field. The involvement of JNK has become clear, yet how it integrates with other pathways or genes that are differentially expressed in the blastema is unknown. Furthermore, the regulation conferring the plasticity of chromatin states necessary for cellular reprogramming depends on the PcG/TrxG system, but little is known about the mechanisms ensuring a tightly regulated response at the chromatin level. Transdetermination studies have also contributed to our current understanding of regeneration, and similarities to human metaplasias have also been suggested since in both cases there are changes to the determined state of epithelial tissues that can be precursors to cancer (Maves and Schubiger 1999). It is, therefore, interesting to consider some similarities between regenerative responses and the early stages of tumor formation. It is plausible that damaging events in tissues initiate a regenerative response but lead to an unfaithful outcome if the route to the developmental program of choice is lost as a result of altered cellular plasticity. Regeneration and tumorigenesis could, therefore, be regarded as two sides of the same coin where the derailment of a repairing program could turn into a damaging response.

## Beyond physiology: imaginal discs as tumor models

The normal functions of many genes identified as mutated in human cancers were first described in flies, and thus, *Drosophila* research has contributed substantially to our understanding of tumor biology. These genes include several components of the conserved signaling pathways mentioned thus far and also tumor suppressors like p53, NF1, APC, and Rb. There are also examples of more direct contributions, where mutations lead to tumor formation in flies and have thus become known as fly tumor models. Interestingly, flies may also spontaneously develop tumors (found mostly in the testis and gut), and similar to humans, incidence increases with age (Salomon and Jackson 2008). Considering the short life expectancy, these observations might be unexpected but revealed the existence of tumors in normal fly strains. Here, however, the focus will be on tumors resulting from genetic manipulations, which can be informative for cancer biology across species.

It has become clear that many human tumors result from one or more clonal events where cells lose their normal function and start overproliferating, often compromising the interactions with neighboring cells or the microenvironment. The initial steps that abnormal cells require to compromise homeostasis in their native tissue are poorly understood, since these early stages are not clinically accessible. Fortunately, *Drosophila* offers a significant advantage in this regard, where the behavior of only a few cells can be followed after inducing a trigger. In fact, the phenomenon of cell competition, which involves differential cell behavior within a tissue, has been observed and well documented for more than four decades in flies. The discovery of cell competition in *Drosophila* established a remarkable new concept to describe the survival and proliferation of cells with higher fitness at the expense of weaker neighbors. It was first observed in mosaics where heterozygous *Minute* (*M*) cells are eliminated when surrounded by wild-type cells (Morata and Ripoll 1975). *M* encodes a ribosomal component and *M* homozygous mutant cells are lethal, but heterozygous animals are viable and cell competition only occurs when clones of cells with different fitness arise in close proximity (Johnston 2009; Martín et al. 2009; Morata and Ripoll 1975). Additional stimuli can also trigger cell competition, such as different doses of *myc*, *yorkie*, and others. Furthermore, cells seem to assess their fitness in a context-dependent manner (Vincent et al. 2013). For example, cells carrying only one copy of *myc* (*myc/+*) are outcompeted by the surrounding wild-type cells. However, cells that carry an extra copy of *myc* (*myc*(+)) can also outcompete wild-type cells, having become known as “supercompetitors” (de la Cova et al. 2004; Moreno and Basler 2004). Interestingly, the involvement of *myc* in cell competition has recently been described in the early mouse embryo as well, suggesting conserved features in mammals (Clavería et al. 2013). It is important to note that cell competition has direct implications in normal development, namely in growth regulation and maintenance of tissue homeostasis. Despite being a general phenomenon occurring in multicellular organisms, this process parallels several features that are required for cancer formation, where weaker cells are actively eliminated in response to signals originating from fast-growing cells that can take over more space to proliferate (Wagstaff et al. 2013).

In *Drosophila*, tumors have generally been categorized into hyperplastic or neoplastic (Bilder 2004; Gonzalez 2013). While imaginal discs carrying hyperplastic tumors show extensive overproliferation, they maintain relatively normal tissue organization with cells in a monolayer and can often differentiate into adult tissues. On the other hand, neoplastic tumors are more aggressive and the overproliferating cells lose their epithelial architecture. Forming multi-layers, these cells are able to grow uncontrollably and have limited differentiation capacity. The isolation of the first mutation causing tumorigenesis, *lethal giant larvae* (*lgl*), dates back to Bridges, but its malignant properties (cells growing rapidly and invasively, killing their host) were only reported in the 1960s. Mutations in two other genes, *discs large* (*dlg*) and *scribble* (*scrib*), were later identified to also lead to neoplastic tumors (Bilder and Perrimon 2000; Stewart et al. 1972). The three genes share phenotypic similarities where homozygous mutant larvae survive but keep growing as L3 for several days without reaching the pupal stage. Larvae size increases dramatically resembling “giant larvae,” and finally will kill the host. These genes have become known as neoplastic tumor suppressor genes (nTSG).

**Fig. 6:**
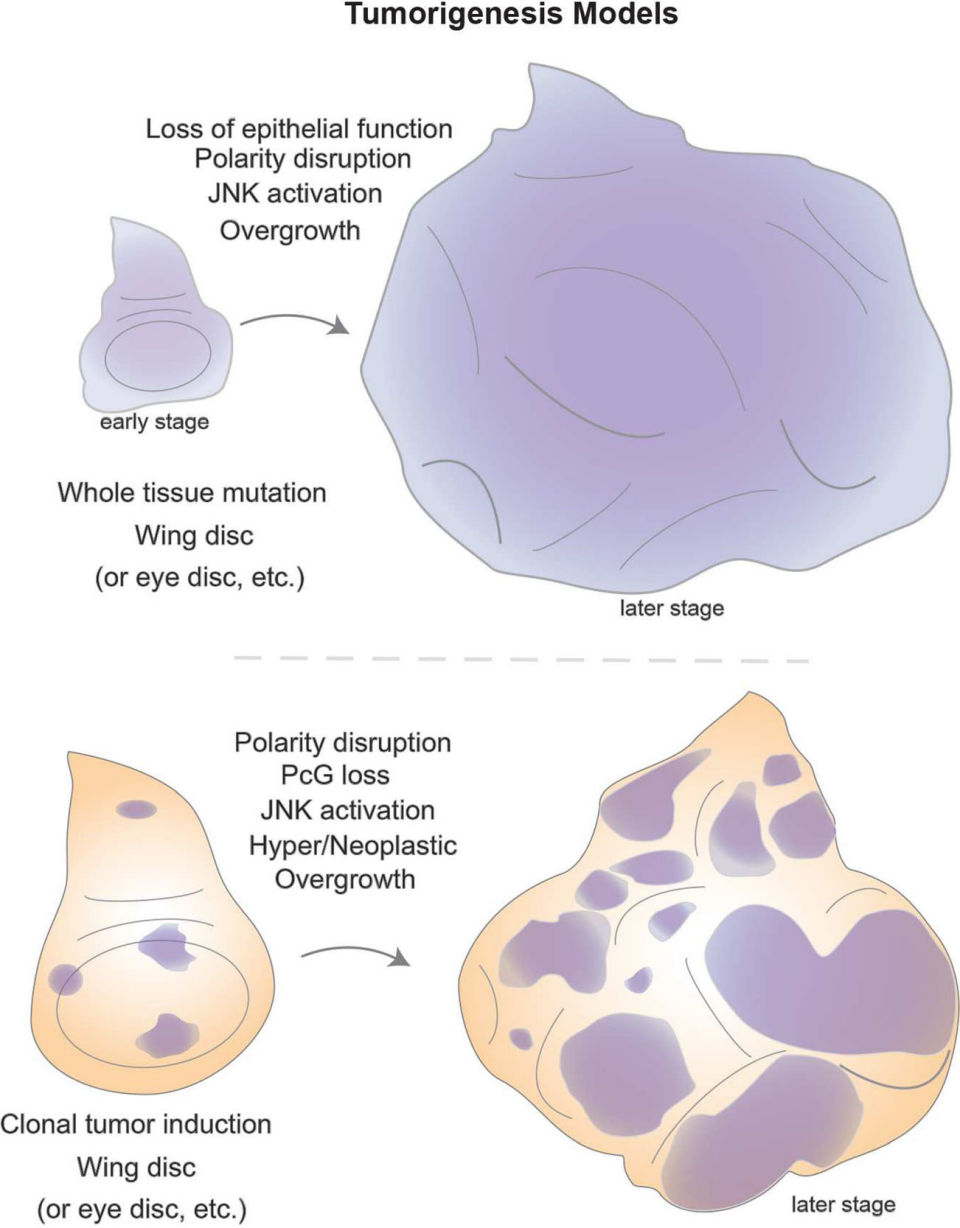
Tumor models in *Drosophila* epithelial tissues. Powerful genetic screens revealed mutations in genes involved in tumorigenesis, namely some classes affecting the development of imaginal discs. Classical mutations like *lgl* (*lethal giant larvae*) displayed abnormal disc development where all tissues of homozygous individuals were affected (*top*). As larval development progresses, it becomes clear that the epithelial structure of disc tissues is aberrant with architectural defects leading to hyperplastic or neoplastic growth. Additional genes were later found to display similar phenotypes and share molecular functions as components of the apico-basal polarity machinery. The combination of mosaic analysis with gain- or loss-of-function experiments provided alternative means to fine-tune screening approaches. Clonal analysis (*bottom*) enables mutant patches to grow surrounded by wild-type tissue, and thus, interactions between tumor and neighboring normal tissue can occur. These approaches revealed, in some instances, that cooperative interactions between conserved oncogenes and polarity determinants enhanced a tumorigenic phenotype in comparison to the effect of each component individually. On the other hand, mutations in epigenetic components (like *ph*, a Polycomb Group member) display strong neoplastic phenotypes in clones that also display abnormal epithelial architecture. Furthermore, the involvement of key signaling pathways is a common feature among fly epithelial tumors, suggesting that signaling events and potentially the interactions between healthy and tumor cells are likely to play a role in the disruptive events leading to tumor formation

Hyperplastic mutations exhibit uncontrolled proliferation, yet tissue architecture and differentiation capacity are still maintained. These phenotypes can be caused by inactivation of genes regulating cell growth, proliferation, or cell death, including many of the genes previously mentioned to regulate growth in a normal context, such as *salvador*, *warts*, *hippo*, *fat* (an atypical cadherin), *expanded*, *Tsc1/2*, *PTEN*, or *Csk* (for details, see (Brumby and Richardson 2005; Hariharan and Bilder 2006; Harvey et al. 2013; Vidal and Cagan 2006)). More invasive tumors, in the neoplastic category, display cells that form rounded cyst-like patches, having lost the ability to maintain an epithelial monolayer crucial for tissue architecture and being unable to differentiate (Fig. 6). A common denominator was identified among *lgl*, *dlg*, and *scrib* in that the proteins they encode interact with the cytoskeleton and define the apico-basal polarity that underlies epithelial cell function (Bilder et al. 2000). Therefore, cell polarity and architecture lie at the core of healthy physiology in epithelia, the tissue type where many human cancers also arise.

Identification of additional neoplastic tumor suppressor genes was facilitated by clonal analysis, as these mutations are lethal at earlier stages and larvae do not survive as long as for lgl/*dlg*/*scrib*. Such nTSGs include *avalanche*, *Rab5*, *tsg101*, and *vps25* which play roles in endocytosis (Lu and Bilder 2005; Moberg et al. 2005; Thompson et al. 2005; Vaccari and Bilder 2005; Wucherpfennig et al. 2003). In these mutants, inactivation of components of the endocytic machinery or ESCRT complexes compromise trafficking between different cellular compartments. There is a shared link between the endocytic and polarity-associated mutants, since failures in endocytosis lead to inappropriate accumulation of apical and basal proteins or signaling receptor molecules, which can mimic phenotypes of polarity nTSGs (Bilder 2004).

Although animals that are entirely mutant for nTSGs display giant larvae phenotypes, it became clear that *scrib* mutant clones could not outcompete their wild-type neighbor cells. Instead, these clones are progressively eliminated in a JNK-dependent manner but can still become tumorigenic if JNK signaling is blocked (Brumby and Richardson 2003). Clonal studies, therefore, uncovered a cooperative effect between disruption of epithelial integrity and oncogenes, such as activated forms of Ras (*ras*^*V12/act*^) and also *Notch*^*act*^. Although *ras*^*act*^ clones alone do not lead to neoplastic tumors (they result in overgrowth), combination with *scrib* clones leads to neoplastic phenotypes (Brumby and Richardson 2003). These observations prompted MARCM genetic screens in the eye disc to identify other factors involved in oncogenic cooperativity (both with *ras*^*act*^ and *Notch*^*act*^). Examples include cytoskeletal components like *rho*/*rhoGEF* or the transcription factor *abrupt* (Brumby et al. 2011; Turkel et al. 2013). These tumors also display a metastatic behavior, as some cells can invade the ventral nerve cord and distant portions of the central nervous system (Pagliarini and Xu 2003).

A common denominator of neoplastic tumors is ectopic stress signaling through JNK activation. Constitutive Ras signaling allows cells to survive by evading JNK-mediated apoptosis (Brumby and Richardson 2003). The invasiveness of *scrib/ras*^*act*^ clones depends on transcriptional activation of *mmp1* in a JNK-dependent manner, and changes in E-cadherin expression were also reported. These findings evoke a possible parallel with evidence from human tumors, which frequently have E-cadherin mutations, and the phenotypes of cancer tissue are reminiscent of alterations known as the epithelial-mesenchymal transition (EMT) (Harvey et al. 2013; Igaki et al. 2006; Uhlirova and Bohmann 2006). Interestingly, neighboring *scrib/ras*^*act*^ clones can actively cooperate and metastasize, by forming interclonal tumors that seem to rely on loss of *scrib* mostly during the early steps, as in later stages clones are mostly composed of *ras*^*V12*^-expressing cells (Wu et al. 2010). JAK-STAT activity has also been implied as an oncogenic driver in *ras*-mediated tumors. Overall, several aspects of these tumors exhibit features reminiscent of cell competition, hinting that it will be interesting to further investigate the parallels between competitive interactions in regulating normal growth or upon losing the ability to control it, leading to tumors (Levayer et al. 2015; Vincent et al. 2013).

Disruption of key chromatin regulators has also been shown to lead to tumorigenesis. Loss of *polyhomeotic* (*ph*), a component of the Polycomb Repressive Complex 1 (PRC1), in eye disc clones results in massive overgrowth of eye and antennal tissue (Classen et al. 2009; Martinez et al. 2009). Clones induced by the MARCM technique (Fig. 4) display a severely disorganized architecture, loss of polarity, and some metastatic properties as well as aberrant signaling activities (Fig. 6). These tumors are dependent on JAK-STAT and Notch signaling activities, and have also been reported to lead to massive overproliferation in an RNAi screen for Notch regulators in the wing disc (Saj et al. 2010). Blocking these pathways ameliorates tumor burden, suggesting that the derailment of signaling pathways underlies a significant effect on the tumor phenotype (Feng et al. 2011, 2012). An overexpression screen for modifiers of *Notch*^*act*^ tumors uncovered a hit, named *eyeful*, which mapped within two genes (*pipsqueak* and *longitudinals lacking*) that produce transcription factors involved in the recruitment of Polycomb and chromatin complexes to promoter regions (Vallejo and Gutierrez-Aviño 2006).

Globally, fly tumor models display several phenotypes that parallel human cancer hallmarks, such as the ability to sustain overproliferation and growth, the capacity to evade apoptotic signals, the inability to respond to anti-proliferative signals, and the capability to metastasize to sites distant from their origin (Brumby and Richardson 2005; Gonzalez 2013). The use of transplantation techniques, pioneered several decades ago, became again instrumental to demonstrate the unlimited growth and survival of fly tumors in hosts, analogous to routine allograft experiments in mice (Rossi and Gonzalez 2015). It was demonstrated, for example, that ph tumor tissue does not accumulate genome instability even after prolonged cultivation. This suggests that an epigenetic derailment of gene expression control is sufficient to maintain neoplastic growth (Sievers et al. 2014). There are, however, important limitations in that certain facets of human tumorigenesis are missing, since flies have a more rudimentary immune system and lack a closed circulatory system. These aspects restrict some direct comparisons with human cancer since angiogenesis and dedicated tumor immunological responses would not be easily modeled in flies. Nonetheless, the great advantages of fly research remain at the forefront in identifying the basic molecules that may have conserved roles which would otherwise be very difficult to find directly in mammals.

Namely, *Drosophila*’s ability to contribute to the early steps of tumorigenesis has great potential, not only from the examples described here but also from emerging knowledge resulting from this very active research field. Many initial cancer triggers remain a mystery, which could be revealed from studies concerning key aspects of the observed fly tumor phenotypes. Moreover, it will be interesting to further explore the links between disrupted polarity, a common feature, as well as ectopic activation of several signaling cascades. An important open question entails how such pathways may influence the regulation of gene expression through transcription factors or chromatin regulators that exert global switches. These can, in a controlled fashion (like the regenerative process), result in faithful tissue organization, but when impaired, they may redefine cellular identities in a pathological manner.

## Conclusions and perspectives

Imaginal discs are the Swiss army knife in *Drosophila* research, serving as versatile experimental systems to address a variety of scientific topics in cell and developmental biology. Here, we have covered a broad range of topics where the use of imaginal discs supported discoveries in cell and developmental biology, revealing conserved pathways and mechanisms. The use of these epithelial precursor organs has enabled functional studies in the context of a developing organism, benefitting from the implementation of genetic tools that considerably contributed to elucidating conserved molecular mechanisms.

With the emergence of new methods and technologies, the potential of imaginal discs as experimental systems remains unchallenged. The combination of established sophisticated tools with modern techniques promises to yield further contributions in developmental and cell biology. Research topics at the interface of physics and biology, like tissue mechanics, are having considerable progress in recent years by using imaginal discs to examine forces and cell interactions in their native tissues of origin. The improvement of long-term in vitro disc culture methods promises to bring many benefits to this field, and combination with other techniques such as live imaging, optogenetics, genome engineering (e.g., CRISPR/Cas9 coupled to homologous recombination), or tissue-specific protein-targeting nanobodies is expected to enable novel and cross-disciplinary discoveries that are yet unforeseen.

Despite the minute size of imaginal structures within the small fly, their contribution to answering fundamental questions has been immense, leading to a far-reaching insight across species. The conservation of many fly genes, proteins, and processes, together with developmental concepts common to all organisms, will provide profound implications for a better understanding of human development, physiology, and biomedicine going beyond measure.
